# Role of NAD^+^-Dependent Malate Dehydrogenase in the Metabolism of *Methylomicrobium alcaliphilum* 20Z and *Methylosinus trichosporium* OB3b

**DOI:** 10.3390/microorganisms3010047

**Published:** 2015-02-27

**Authors:** Olga N. Rozova, Valentina N. Khmelenina, Ksenia A. Bocharova, Ildar I. Mustakhimov, Yuri A. Trotsenko

**Affiliations:** 1 Laboratory of Methylotrophy, Skryabin Institute of Biochemistry and Physiology of Microorganisms, RAS, Prospect Nauki 5, Pushchino 142290, Russia; E-Mails: olgan.rozova@gmail.com (O.N.R.); trotsenko@ibpm.pushchino.ru (Y.A.T.); 2 Department of Microbiology and Biotechnology, Pushchino State Institute of Natural Sciences, Prospect Nauki 3, Pushchino 142290, Russia; E-Mails: ksenia.alpha4160@mail.ru (K.A.B.); mii80@rambler.ru (I.I.M.)

**Keywords:** l-malate dehydrogenase, catalytic efficiency, thermostability, tricarboxylic acid cycle, methanotrophs, *Methylomicrobium alcaliphilum*, *Methylosinus trichosporium*

## Abstract

We have expressed the l-malate dehydrogenase (MDH) genes from aerobic methanotrophs *Methylomicrobium alcaliphilum* 20Z and *Methylosinus trichosporium* OB3b as his-tagged proteins in *Escherichia coli*. The substrate specificities, enzymatic kinetics and oligomeric states of the MDHs have been characterized. Both MDHs were NAD^+^-specific and thermostable enzymes not affected by metal ions or various organic metabolites. The MDH from *M. alcaliphilum* 20Z was a homodimeric (2 × 35 kDa) enzyme displaying nearly equal reductive (malate formation) and oxidative (oxaloacetate formation) activities and higher affinity to malate (K_m_ = 0.11 mM) than to oxaloacetate (K_m_ = 0.34 mM). The MDH from *M. trichosporium* OB3b was homotetrameric (4 × 35 kDa), two-fold more active in the reaction of oxaloacetate reduction compared to malate oxidation and exhibiting higher affinity to oxaloacetate (K_m_ = 0.059 mM) than to malate (K_m_ = 1.28 mM). The k_cat_/K_m_ ratios indicated that the enzyme from *M. alcaliphilum* 20Z had a remarkably high catalytic efficiency for malate oxidation, while the MDH of *M. trichosporium* OB3b was preferable for oxaloacetate reduction. The metabolic roles of the enzymes in the specific metabolism of the two methanotrophs are discussed.

## 1. Introduction

The malate dehydrogenase (MDH, l-malate: NAD oxidoreductase, EC 1.1.1.37) catalyzing the NAD(P)^+^/NAD(P)H-dependent interconversion of l-malate to oxaloacetic acid (OAA) is widespread in the three domains of life. It plays crucial roles in many metabolic pathways, including the tricarboxylic acid (TCA) cycle, energy generation and the formation of metabolites for biosynthesis.

Aerobic bacteria utilizing methane as a sole source of carbon and energy (methanotrophs) belong to the Alpha and Gamma classes of Proteobacteria and the phylum Verrucomicrobia [1,2]. Since these bacteria are able to obtain energy from the oxidation of reduced C_1_ compounds, the TCA cycle would not be an obligatory way for energy generation. The complete oxidative TCA cycle could function in the alphaproteobacterial methanotrophs, such as *Methylosinus trichosporium* OB3b, assimilating carbon via the serine pathway with the formation of C_2_ and C_3_ compounds as primary intermediates; however, the activity of 2-oxoglutarate dehydrogenase was rather low [3]. The gammaproteobacterial methanotrophs, such as *Methylomicrobium alcaliphilum* 20Z, use the ribulose monophosphate (RuMP) cycle of C_1_-assimilation, producing hexose phosphates as the first intermediates. In spite of the presence of the 2-oxoglutarate dehydrogenase genes in their genomes, the activity of the enzyme has never been demonstrated [4,5,6]. Although the role of the TCA cycle in obligate methanotrophic bacteria is supposed to be more anabolic than catabolic, it still remains controversial. An important TCA cycle enzyme, MDH, has never been characterized in these bacteria. Due to the ability to convert methane into multicarbon compounds, methanotrophs have attracted attention as agents for mitigating methane emissions and for biotechnological applications [7]. Investigation of the key metabolic pathways in *M. trichosporium* OB3b and *M. alcaliphilum* 20Z, the model methanotrophic species, should contribute to metabolic engineering for the development of processes for methane-based biocatalysis in the future. Since MDH is widely used in enzymatic analysis and NAD(H) regeneration, the study of methanotrophic MDH might provide basic information for its utilization.

In this study, the recombinant MDHs from *M. trichosporium* OB3b and *M. alcaliphilum* 20Z have been purified and characterized for the first time. We have shown that the biochemical properties of MDHs are appropriate for the specific metabolism of these bacteria possessing complete or uncompleted TCA cycles.

## 2. Materials and Methods

### 2.1. Bacteria and Growth Conditions

*M. alcaliphilum* 20Z (VKMB-2133, NCIMB14124) and *M. trichosporium* OB3b (ATCC 35070) were grown in a nitrate mineral salt medium P under methane-air atmosphere (1:1) at 30 °C [8,9]. The medium for *M. alcaliphilum* 20Z cultivation was additionally supplied with 3% NaCl and Na-carbonate buffer at a final concentration 0.1 M for pH adjustment (pH 9.0). *Escherichia coli* strain BL21 (DE3) (Novagen) was grown at 37 °C in a selective Luria–Bertani (LB) agar or broth under continuous shaking (150 rpm). Ampicillin (100 μg/mL) was added for the growth of plasmid-bearing *E. coli* cells.

### 2.2. DNA Manipulations

Plasmid isolation, digestion by restriction enzymes, agarose gel electrophoresis, ligation and transformation of *E. coli* cells were performed according to Sambrook and Russell [10]. Restriction enzymes, T4 DNA-ligase, *Pfu* DNA-polymerase, a dNTPs mixture and Page Ruler Prestained Protein Ladder for SDS-PAGE, were purchased from Thermo Scientific (Lithuania).

### 2.3. Expression of the Mdh Genes and Purification of Recombinant MDHs

Chromosomal DNAs from *M. alcaliphilum* 20Z and *M. trichosporium* OB3b were prepared as described previously [11]. The *M. alcaliphilum mdh* gene (CCE24885) was amplified by PCR using the primers designed from the sequence available in GenBank (Accession Number NC_016112): forward (5′-TCCATATGAAAACGCCAGTTAAAATTGCC) and reverse (5′-TACTCGAGGAGCAAATGCTTTACCGCTTCGC) containing recognition sites for the *Nde*I and *Xho*I restriction endonucleases, respectively. The following primers were used for amplification of the *mdh* gene (WP_003612980) from the DNA of *M. trichosporium* OB3b (Accession Number ADVE00000000): forward (5′-TACATATGATGGCGCGCAAGAAAATCGCA) and reverse (5′-TACTCGAGGGCGAAAGACGGGTCGAGCG), containing recognition sites for the *Nde*I and *Xho*I endonucleases, respectively. For the expression of the *mdh* genes, the vectors pET22b:mdh-20Z or pET22b:mdh-OB3b were transformed into *E. coli* BL21 (DE3). The transformed *E. coli* cells were grown at 37 °C in a liquid LB medium containing 100 μg/mL ampicillin; the *mdh* expression was induced by adding 0.5 mM isopropyl-1-thio-β-D-galactopyranoside (IPTG) at OD_600_ of 0.6–0.8. After 15 h of growth at 18 °C, the cells were harvested by centrifugation (30 min at 8 °C and 5000× *g*) and stored at −20 °C. The MDH-His_6_-tag proteins were purified by affinity chromatography on a Ni^2+^-nitrilotriacetic acid (Ni-NTA) column as described earlier [12], and enzyme purity was analyzed by 12% SDS-PAGE. The purified enzymes were stored in 40% glycerol at −20 °C.

### 2.4. Determination of MDH Molecular Masses

The quaternary forms of the enzymes were analyzed by non-denaturating gel electrophoresis and gel filtration methods. The gel electrophoresis was carried out by using pore-limited gradient polyacrylamide (4%–30%) [13]. Gel filtration was carried out on an accurate Sephacryl S-200 (Sigma-Aldrich, Schnelldorf, Germany) column equilibrated by mobile phase buffer consisting of 20 mM Tris-HCI pH 8.0 and 100 mM NaCI. The reference proteins ferritin (440 kDa), amylase (200 kDa), alcohol dehydrogenase (150 kDa), bovine serum albumin (BSA, 66 kDa) and equine myoglobin (17 kDa) were obtained from Sigma-Aldrich (Schnelldorf, Germany).

### 2.5. Enzyme Assays

The MDH activity of *M. trichosporium* in the direct reaction (OAA formation) was determined by measuring NAD^+^ recovery at 30 °C in a standard reaction mixture (1 mL) containing 100 mM glycine–NaOH buffer (pH 9.5), 0.17 mM NAD^+^ and ~250 μg MDH-OB3b. The activity of *M. alcaliphilum* MDH was measured at pH 10.0 and ~740 μg MDH-20Z. The reaction was initiated by adding 1 mM malate. The MDH activities in the reverse direction (malate formation) were measured at the optimal pH in the presence of 0.25 mM NADH_2_, and the reactions were initiated by 1 mM OAA.

The following buffers were used to study the pH dependence of MDH activity (100 mM): glycine–NaOH (9.0–10.5), Tris–HCl (pH 7.6–8.9) and K–phosphate (6.0–8.0). Pyruvate, phosphoenolpyruvate, ATP, ADP, AMP, glucose-1-phospate, fructose-1-phosphate, fructose-1,6-bisphosphate, fructose-6-phosphate, ribose-1-phosphate, ribose-5-phosphate (at a concentration of 5 mM), glycerate, lactate, α-ketoglutarate, citrate, serine, pyrophosphate (1 mM) or КH_2_PO_4_ (10 mM) were tested as potential effectors. To test the effect of divalent metals on the MDH activity, aquatic stock solutions of CuCl_2_, MgCl_2_, MnCl_2_, CoCl_2_, BaCl_2_, ZnCl_2_ or CaCl_2_ were added to a final concentration of 0.1–1 mM. The effects of different concentrations of NaCl or KCl (0.1, 0.5 and 1 M) were also tested. To determine the thermal stability of MDHs, the aliquots of the enzymes in Eppendorf tubes were incubated from 5 min to 3 h at 30, 40, 50, 60 and 70 °C. The tubes were rapidly cooled in an ice bath, and residual MDH activity was determined at 30 °C. The percentage of residual activity was calculated by comparison with the non-incubated enzyme. The optimal temperature for MDH activities was tested in the reaction at 10–70 °C. The kinetic parameters (V_max_ and K_m_) were determined by using different concentrations of one substrate and a constant concentration of the other substrate in the reaction. The K_m_ and V_max_ values were calculated using SigmaPlot (version 10, Systat Software, San Jose, CA, USA). Protein concentrations were assayed by the Lowry method using BSA as a standard. NADH oxidation/formation rates were followed at 340 nm with a UV-1700 spectrophotometer (Shimadzu, Japan).

### 2.6. Sequence Analysis

The sequences from the NCBI database were obtained by BLAST searches. The alignments of amino acid sequences of different MDHs were generated with ClustalX2 [14]. Minor manual corrections of the alignments were performed. Phylogenetic analysis was performed using MEGA 6 and the neighbor-joining model [15]. There were 343 informative positions in the final dataset.

## 3. Results

### 3.1. Expression of the Mdh Genes and Purification of MDHs

**Figure 1:**
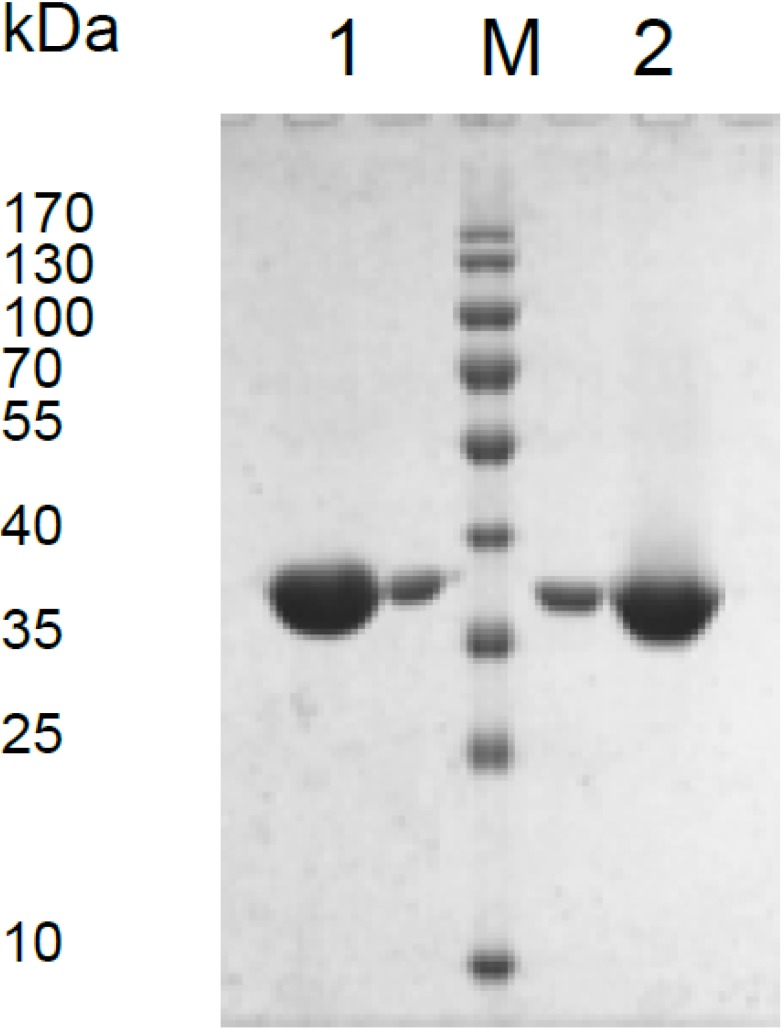
SDS-PAGE of malate dehydrogenase (MDH) from *M. alcaliphilum* 20Z (1) and *M. trichosporium* OB3b (2). M, markers.

Open reading frames annotated as the MDH encoding genes in the genomes of *M. alcaliphilum* 20Z and *M. trichosporium* OB3b were successfully expressed in *E. coli* BL21 (DE3), and the His_6_-tagged proteins were purified from crude extracts of *E. coli* cells by single-step metal-chelating affinity chromatography. Approximately 5–6 mg of each recombinant MDH was purified per 100 mL of culture. The recombinant enzymes catalyzed the NAD^+^-dependent oxidation of malate and the NADH-dependent reduction of OAA. SDS/PAGE of each MDH-His_6_ showed single bands of about 35 kDa, which was in good agreement with the theoretically calculated subunit sizes of 35.8 kDa for the *M. alcaliphilum* MDH and 33.2 kDa for the *M. trichosporium* MDH (Figure 1). According to the native gradient electrophoresis and gel filtration (Figure S1), the *Mr* of the *M. alcaliphilum* MDH (70 kDa) implies that the enzyme exists as a homodimer, whereas the *Mr* of the *M. trichosporium* MDH (140 kDa) indicates its homotetrameric structure. Most of the characterized bacterial MDHs are also NAD^+^-dependent enzymes organized as homodimers or homotetramers [16,17].

### 3.2. Catalytic Properties of Recombinant MDHs

**Figure 2:**
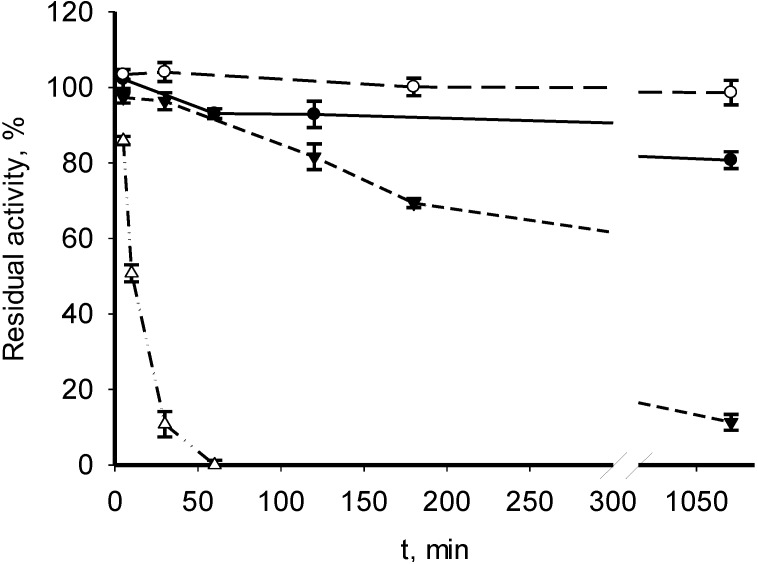
Effect of temperature on the stability of the recombinant MDH from *M. alcaliphilum* 20Z. The enzyme was incubated at 30 (○), 40 (●), 50 (▼) or 60 °C (Δ), and the residual activity was measured at 30 °C.

**Figure 3:**
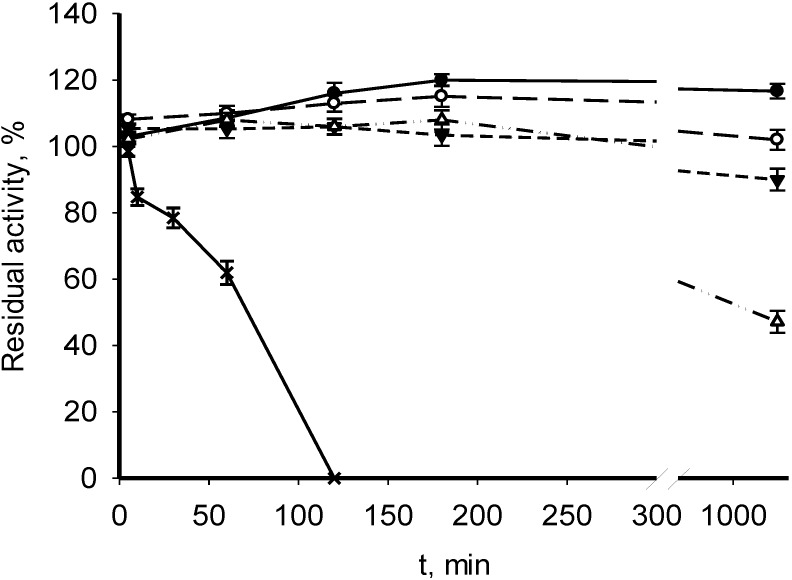
Effect of temperature on the stability of the recombinant MDH from *M. trichosporium* OB3b. The enzyme was incubated at 30 (●), 40 (○), 50 (▼), 60 (Δ) or 70 °C (×), and the residual activity was measured at 30 °C.

The recombinant MDHs from *M. trichosporium* OB3b and *M. alcaliphilum* 20Z were active in a wide pH range (pH 7.5–10.0) in both OAA reduction and malate oxidation. The *M. trichosporium* MDH exhibited maximal activity at pH 9.5, and the *M. alcaliphilum* enzyme 20Z showed a more alkaline optimum (pH 10.0) (Figure S2). The activities of MDHs from both methanotrophs increased up to 60 °С and then gradually decreased (Figure S3). Interestingly, the MDH from *M. alcaliphilum* 20Z fully retained activity after 18-h exposure at 30–40 °С and had a 10% residual activity after 18-h exposure at 50 °С. The enzyme lost 50% of its activity after 10-min heating at 60 °С (Figure 2). The *M. trichosporium* MDH was active in a broader temperature range (Figure 3). It retained full activity after 18-h exposure at 30–60 °С, exhibiting a 1-h half-life time at 70 °С.

The substrate dependence of the activities of MDHs from both methanotrophs in both directions obeyed the Michaelis–Menten kinetics. At 30 °С and the optimal pH, the apparent K_m_ values for the *M. trichosporium* MDH were as follows: 0.059 ± 0.007 mM (ОАA), 0.0376 ± 0.0046 mM (NADН_2_), 1.28 mM ± 0.17 mM (malate) and NAD^+^ 0.33 ± 0.04 mM. The activity of the OAA reduction was two-fold higher than that of malate oxidation (187.81 ± 5.24 and 78.2 ± 3.5 U/mg, respectively). The calculated k_cat_/K_m_ ratios indicated that the enzyme from *M. trichosporium* OB3b had an about 50-fold preference for OAA reduction (k_cat_/K_m_ = 19,344) over l-malate oxidation (k_cat_/K_m_ = 403) (Table 1). Therefore, the MDH from *M. trichosporium* OB3b had a markedly high catalytic efficiency for malate production.

On the contrary, the *M. alcaliphilum* MDH had almost equal activities in both directions (15.05 U/mg of protein for OAA reduction and 20.75 U/mg of protein for malate oxidation). The apparent K_m_ values of the enzyme measured at 30 °С and pH 10 were as follows: 0.34 ± 0.03 mM (OAA), 0.0252 ± 0.0004 mM (NADH_2_), 0.11 ± 0.01 mM (malate) and 0.45 ± 0.08 mM (NAD^+^). Thus, the enzyme from *M. alcaliphilum* displayed higher affinity towards malate than OAA. Analysis of the k_cat_/K_m_ ratios showed that the enzyme had an about two-fold preference for l-malate oxidation (k_cat_/K_m_ = 5648) compared to OAA reduction (k_cat_/K_m_ = 2558). Therefore, MDH had higher catalytic efficiency for OAA production than for malate synthesis.

**Table 1:** Properties of the recombinant malate dehydrogenases from *Methylomicrobium alcaliphilum* 20Z and *Methylosinus trichosporium* OB3b.

Parameter	*M. alcaliphilum* 20Z	*M. trichosporium* OB3b	*Streptomyces coelicolor* A3(2) [18]	*Nitrosomonas europaea* [19]
Subunit molecular mass, kDa	70 (35 × 2)	170 (35 × 4)	73 (36 × 2)	nd
pH opt for malate oxidation	10	9.5	nd	8.5
pH optimum for oxaloacetate reduction	10	9.5	6.5/6.8	8.5
Temperature optimum, °C	60–65	60	30/50	55
K_m_ (mM)				
malate	0.11	1.28	0.494	5
oxaloacetate	0.34	0.059	0.189	0.02
NAD^+^	0.45	0.33	0.15	0.024
NADH_2_	0.025	0.037	0.083	0.022
V_max_ malate oxidation (U/mg)	15	78	4.02	nd *
V_max_ oxaloacetate reduction (U/mg)	21	188	1,600	nd *
k_cat_ malate (1/s)	621	516	471	nd
k_cat_ oxaloacetate (1/s)	870	1141	1870	nd
k_cat_/K_m_ malate (1/s mM)	5648	403	9.53	nd
k_cat_/K_m_ oxaloacetate (1/s mM)	2558	19,344	10,000	nd
Inhibitors	No	No	Zn^2+^, Со^2+^, Fe^2+^	Zn^2+^, Fe^2+^, Mn^2+^
Activators	No	No	No	AMP, Cu^2+^

* The average activities of the partially purified enzyme were: 0.89 mU/mg of protein for malate oxidation and 18 mU/mg of protein for oxaloacetate reduction [19]. nd, not determined.

No essential effect of divalent metal ions on the activities of both MDHs was revealed (Table S1). NaCl or KCl (100 mM) did not inhibit MDH activities. The *M. trichosporium* MDH was more sensitive to high ionic strength *in vitro*, retaining a 37%–40% activity at 1 M NaCl or KCl, compared to the halotolerant *M. alcaliphilum* MDH retaining a 61%–74% activity (Table S1). Pyruvate, phosphoenolpyruvate, citrate, glucose-1-phospate, fructose-1-phosphate, fructose-1,6-bisphosphate, fructose-6-phosphate, ribose-1-phosphate, ribose-5-phosphate, glycerate, lactate, α-ketoglutarate, serine, ATP, ADP, AMP, pyrophosphate or KH_2_PO_4_ were tested as potential effectors, but no allosteric regulator of the two enzymes was found among these compounds. Therefore, the functions of the enzymes in both forward and reverse reactions seemed to be regulated at the level of substrate and product concentrations in the cells.

### 3.3. Phylogenetic Positions of MDHs from M. alcaliphilum 20Z and M. trichosporium OB3b

**Figure 4:**
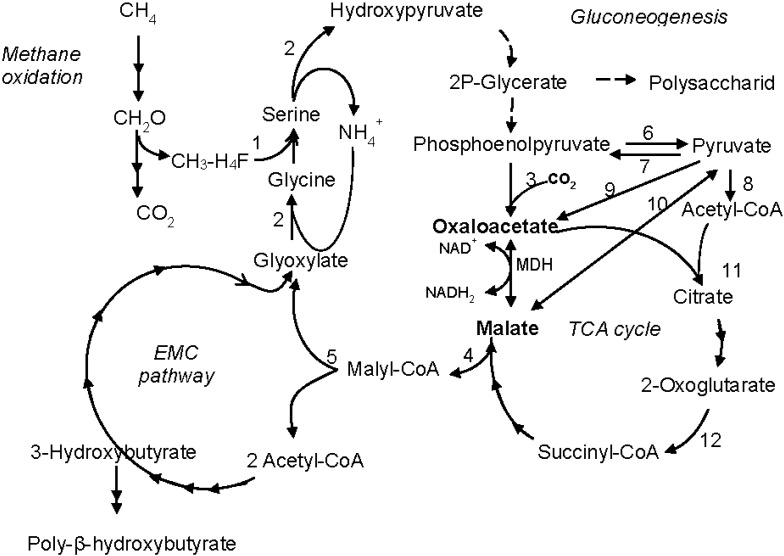
Schematic representation of the central metabolism in *Methylosinus trichosporium* OB3b based on enzymatic data [8,22,23] and analysis of the complete genome sequence. 1, serine hydroxymethyltransferase; 2, serine-glyoxylate transaminase; 3, phosphoenolpyruvate carboxylase; 4, malate thiokinase; 5, malyl-CoA lyase/beta-methylmalyl-CoA lyase; 6, pyruvate kinase; 7, phosphoenolpyruvate synthetase; 8, pyruvate dehydrogenase complex; 9, pyruvate carboxylase; 10, malic enzyme; 11, citrate synthase; 12, 2-oxoglutarate dehydrogenase. EMC, ethylmalonyl-CoA.

The BLAST search of the available database revealed that the MDHs of *M. alcaliphilum* 20Z and *M. trichosporium* OB3b are only distantly related to each other, showing a 17% identity at the amino acid level. They can be affiliated with different clades of the LDH/MDH super-family dehydrogenases according to the ascertained classification [16,20]. The enzyme from *M. alcaliphilum* 20Z belongs to group I of MDHs, which includes the well-described subgroups of dimeric mitochondrial and cytosolic, along with bacterial MDHs [16,21]. It is clustered together with the MDH-like sequences from other gammaproteobacterial methanotrophs (61%–73% identities) (Figure 4), as well as non-methylotrophic representatives of Gamma- and Beta-proteobacteria and the phylum *Deinococcus-Thermus* (*Thermus thermophilus*). The *M. trichosporium* MDH belongs to group III of the LDH-like MDHs presented mostly as homotetrameric enzymes [16]. It shares the highest sequence identity to MDH from other alphaproteobacterial methane and methanol utilizers (78%–85%) (Figure S4). This clade also includes the MDH-like sequences from the verrucomicrobial methanotrophs *Methylacidiphilum infernorum* and *M. fumariolicum* (45% identity with the *M. trichosporium* enzyme).

## 4. Discussion

We have found herewith that the MDHs of *M. alcaliphilum* 20Z and *M. trichosporium* OB3b are highly NAD^+^-specific enzymes, like most bacteria and archaea [17]. Although both methanotrophs are mesophilic, optimally growing at 28 °C, heat inactivation has shown that their MDHs are thermostable similar to those from some other microorganisms. Thermostable properties were demonstrated for the enzyme from thermophilic Vulcanithermus medioatlanticus and *Archaeoglobus fulgidus*, as well as mesophilic *Macromonas bipunctata*, *Rhodopseudomonas palustris*, *Sphaerotilus* sp. D-507, *Streptomyces coelicolor* and *Streptomyces avermitilis* [24,25,26,27,28,29]. We showed here that a relatively high yield of recombinant enzyme could be obtained from two methanotrophs by expression in an *E. coli* host.

The kinetic properties of the two MDHs correlate well with the carbon metabolism of the two methanotrophs. The higher catalytic efficiency (k_cat_/K_m_ ratio) of the *M. trichosporium* MDH for malate synthesis than for OAA production corresponds to the central position of malate in the serine pathway (Figure 4). In this pathway, malate is activated to malyl-CoA, which is cleaved into acetyl-CoA and glyoxylate, the latter being a precursor of glycine, the primary acceptor of formaldehyde. In turn, acetyl-CoA can enter the ethylmalonyl-CoA (EMC) cycle, where another molecule of glyoxylate is formed [22] or directed to the synthesis of polymeric compounds, such as fatty acids and poly-β-hydroxybutyrate (PHB) (Figure 4). The main source of OAA for the MDH reaction may be phosphoenolpyruvate (PEP) carboxylation by PEP carboxylase, which is highly active in the serine pathway methanotrophs [8]. Importantly, the genome of *M. trichosporium* OB3b encodes two PEP carboxylases sharing 33% identity at the amino acid level, which suggests the enzymatic redundancy at the stage of PEP to oxaloacetate conversion [23].

**Figure 5:**
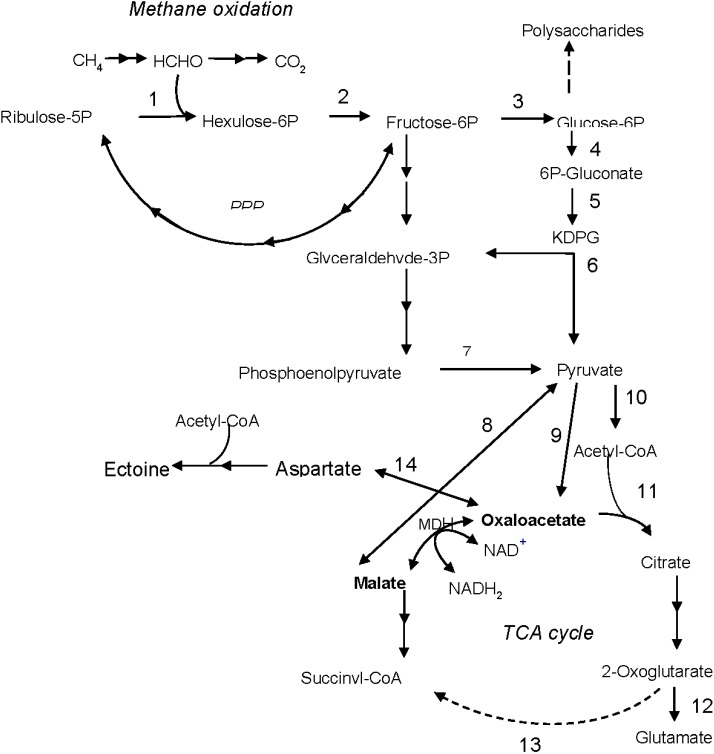
Schematic representation of the central metabolism in *Methylomicrobium alcaliphilum* 20Z based on enzymatic data [5,7,32] and the complete genome sequence. 1, hexulose-6-phosphate synthase; 2, hexulose-6-phosphate isomerase; 3, phosphoglucose isomerase; 4, glucose-6-phosphate dehydrogenase; 5, 6-phosphogluconate dehydrogenase; 6, 2-keto-3-deoxy-6-phosphogluconate aldolase; 7, pyruvate kinase; 8, malic enzyme; 9, pyruvate carboxylase; 10, pyruvate dehydrogenase complex; 11, citrate synthase; 12, glutamate dehydrogenase; 13, putative 2-oxoglutarate dehydrogenase; 14, aspartate aminotransferase. PPP, pentose phosphate pathway.

On the contrary, the k_cat_/K_m_ ratio of the *M. alcaliphilum* MDH indicates that the enzyme has about a two-fold preference for L-malate oxidation (OAA formation). This enzymatic feature is in accordance with the high demand of the halotolerant methanotroph for aspartate, the precursor of the major osmoprotectant ectoine [9,30]. An additional osmolyte in this bacterium is glutamate (its intracellular concentration can reach 0.4 M) [9]. OAA condensation with acetyl-CoA with citrate formation may reinforce the oxidative branch of the incomplete TCA cycle, where 2-oxoglutarate (glutamate precursor) is synthesized. Hence, we may speculate that the MDH of *M. alcaliphilum* 20Z is well-adapted to the halophilic nature of the methanotroph, but additional studies are needed to confirm this hypothesis. In turn, malate for the MDH reaction in *M. alcaliphilum* 20Z may be formed from pyruvate via the putative “malic”-enzyme encoded by its genome (Figure 5).

It should be mentioned that the MDH from *M. alcaliphilum* 20Z shows relatively high sequence identity (55%) with MDHs from the chemoheterotroph *S. coelicolor* and the autotroph *Nitrosomonas europaea*, possessing either a complete (*S. coelicolor*) or incomplete (*N. europaea*) TCA cycle. Despite their affiliation with the same clades of MDHs, they exhibited higher catalytic efficiencies for OAA reduction than for malate oxidation, operating advantageously in the direction of malate synthesis [19,27] (Table 1). Apart from *M. alcaliphilum* 20Z, the genomes of *N. europaea* and *S. coelicolor* code for the PEP carboxylase. It was hypothesized that the MDHs of *N. europaea* and *S. coelicolor* provided the conversion of the product of anaplerotic CO_2_ fixation in the form of OAA to succinyl-CoA and other biosynthetic intermediates [19]. This may be also true for the highly divergent *M. trichosporium* MDH advantageously operating in the direction of malate synthesis. Thus, in both methanotrophs studied, l-malate dehydrogenases are well adapted for their metabolic roles in the specific pathways generating essential biosynthetic metabolites.

Interestingly, the genomes of *M. alcaliphilum* 20Z and three other gammaproteobacterial methanotrophs (*Methylomicrobium buryatense*, *Methylomicrobium album* and *Methylobacter tundripaludum*) possess sequences homologous to the LDH-like MDH (28% identities to *M. trichosporium* MDH) (Figure S4). These sequences have unusual amino acids in the catalytic site at position 102 discriminating the substrate specificity of LDH and MDH enzymes: Thr (*M. alcaliphilum* 20Z), Ser (*M. album*), Met (*M. buryatense*) and Asp (*M. tundripaludum*), instead of conservative Gln in LDH or Arg in MDH [18,31]. Determination of the products of these genes is in progress.

## 5. Conclusions

Overall, we have demonstrated that MDHs from two obligate methanotrophs *M. trichosporium* OB3b and *M. alcaliphilum* 20Z are well-adapted for their role in the specialized metabolism of these bacteria realizing different pathways for carbon assimilation and possessing complete or uncompleted TCA cycles. Although *M. trichosporium* OB3b possesses a complete set of the citric acid cycle enzymes, implying its oxidative function of the TCA cycle, however its MDH in vitro exhibits remarkably higher catalytic efficiency for oxaloacetate reduction than for malate oxidation. The enzyme therefore could be involved in a reductive biosynthetic pathway that allows the products of primary C_1_-assimilation to be converted to malate, a central intermediate of the serine pathway. In contrast, MDH from *M. alcaliphilum* 20Z has preference in oxidative direction (displaying about two-fold higher catalytic efficiency towards malate oxidation over oxaloacetate reduction) which contradicts the established point of view that the TCA cycle in the RuMP-pathway methanotrophs is branching into the oxidative and reductive arms. These catalytic enzyme properties are in accordance with a high demand of the halotolerant bacterium in aspartate as precursor of osmoprotective compounds. In overall, the biochemical properties of two MDHs correspond to the accepted supposition that the TCA cycle in obligate methanotrophs fulfills predominantly anabolic function [3]. The high activity and significant thermostability of the methantrophic MDHs as well as possibility to be obtained as recombinant enzymes, make them applicable enzymatic systems.

## Supplementary Files

Supplementary File 1
