# Supplementary material for: Role of NAD+-Dependent Malate Dehydrogenase in the Metabolism of Methylomicrobium alcaliphilum 20Z and Methylosinus trichosporium OB3b
Source: Microorganisms. 2015 Feb 27;3(1):47–59. doi: 10.3390/microorganisms3010047 (PMC5023229; doi:10.3390/microorganisms3010047)
Supplement: Supplementary File 1 [file microorganisms-03-00047-s001.docx]

**Supplementary Information**

**Table S1.** Effect of mono- and divalent metal on activity of L-malate dehydrogenases from *Methylosinus trichosporium* OB3b and *Methylomicrobium alcaliphilum* 20Z.

| **Ions** | **Concentration (mM)** | **Residual Activity (%)** | |
| --- | --- | --- | --- |
|  |  | ***M. trichosporium* OB3b** | ***M. alcaliphilum* 20Z** |
| Without addition |  | 100 | 100 |
| Na^+^ | 100 | 103 | 98 |
|  | 500 | 64 | 82 |
|  | 1000 | 37 | 61 |
| K^+^ | 100 | 100 | 103 |
|  | 500 | 73 | 98 |
|  | 1000 | 41 | 74 |
| Zn^2+^ | 1 | 102 | 103 |
|  | 0.1 | 101 | 113 |
| Mg^2+^ | 1 | 106 | 118 |
|  | 0.1 | 94 | 108 |
| Co^2+^ | 1 | 94 | 96 |
|  | 0.1 | 100 | 94 |
| Ca^2+^ | 1 | 111 | 112 |
|  | 0.1 | 101 | 103 |
| Cu^2+^ | 1 | 97 | 133 |
|  | 0.1 | 110 | 134 |
| Ba^2+^ | 1 | 104 | 106 |
|  | 0.1 | 101 | 103 |
| Mn^2+^ | 1 | 106 | 103 |
|  | 0.1 | 100 | 115 |

Metals were added as chloride salts. One hundred percent corresponded to activity of either 187 U/mg of protein (*M. trichosporium* OB3b) or 12 U/mg of protein
(*M. alcaliphilum* 20Z) in the direction of OAA reduction.


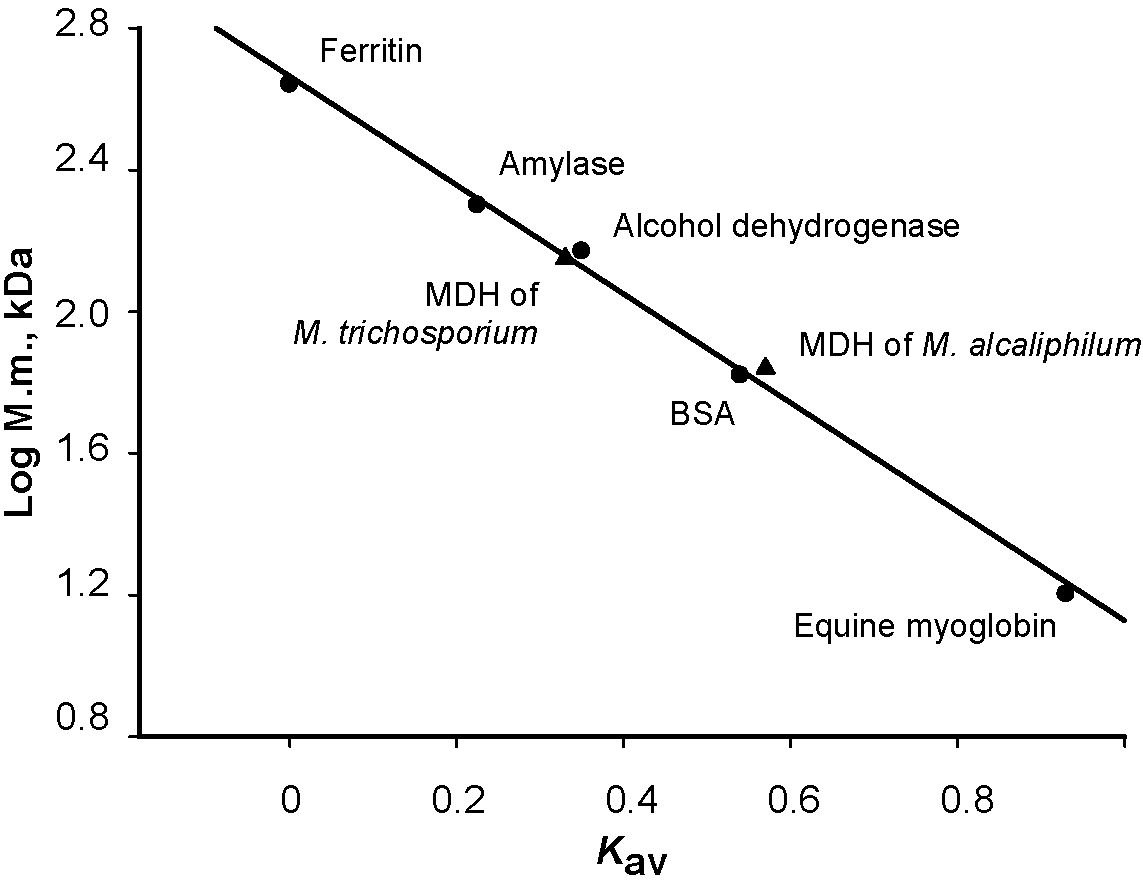


**Figure S1.** Determination of the native molecular mass for the *M. alcaliphilum* and *M. trichosporium* MDHs with Sephacryl S-200 column. K_av_, Relative elution volume.


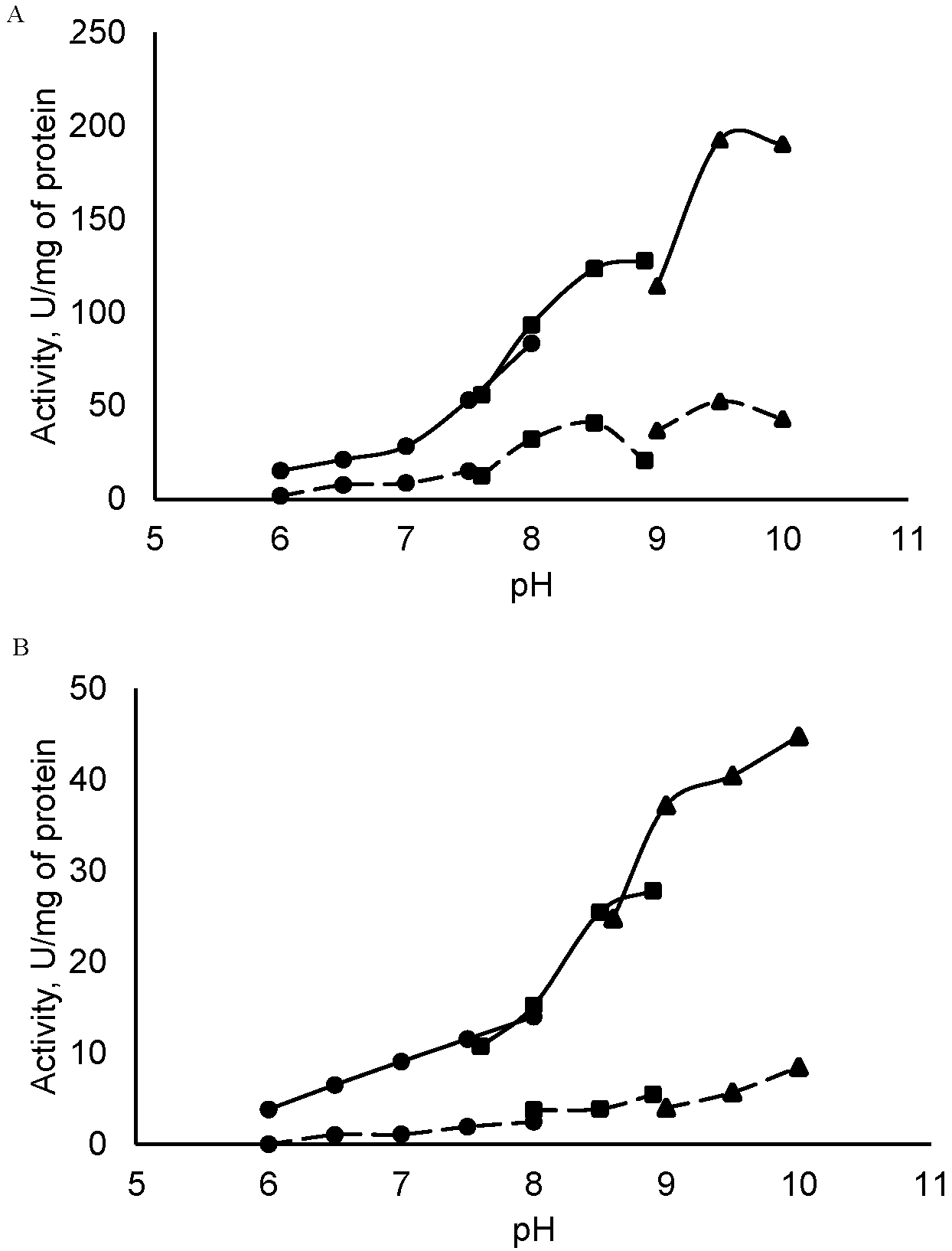


**Figure S2.** Effect of pH on activity of MDH from *M. trichosporium* OB3b (solid line) and *M. alcaliphilum* MDH (dashed line) in the direct (**A**) and reverse (**B**) reactions in ● K-phosphate, in ■ Tris-HCl and ▲ Glycine-NaOH buffers.


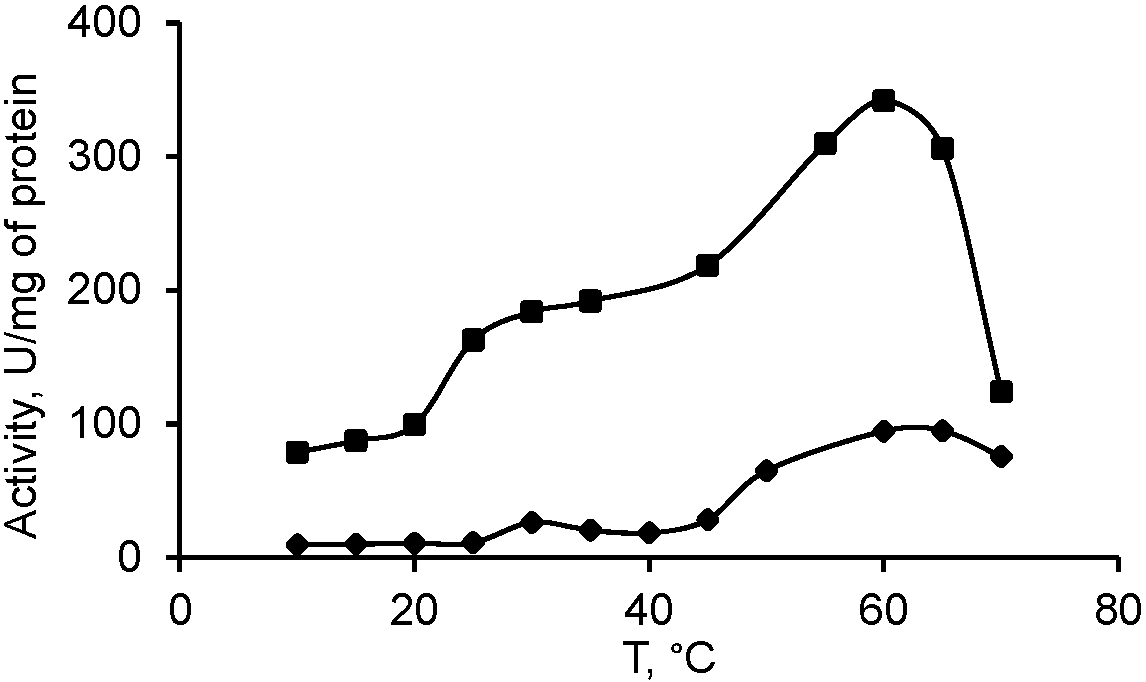


**Figure S3.** Effect of temperature on activity of MDHs from *M. alcaliphilum* (♦) and *M. trichosporium* OB3b (■).


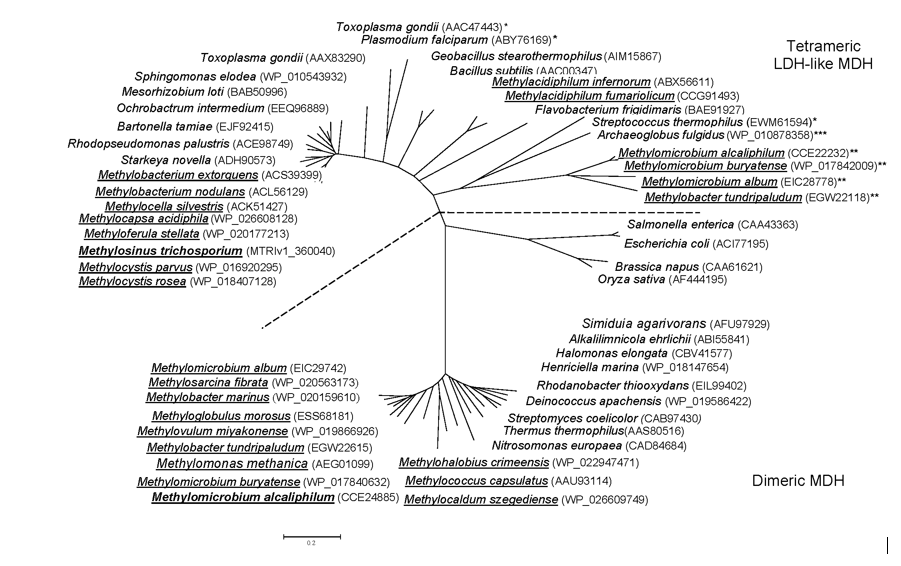


**Figure S4.** Phylogenetic relationships of MDHs from *M. alcaliphilum* 20Z and *M. trichosporium* OB3b to selected MDHs of (micro)organisms. The tree was constructed using the MEGA 6 program (computed from 100 independent trials). Underlined organisms are methane-utilizing bacteria. In brackets the protein sequence accession numbers in the GeneBank are given. The scale bar corresponds to the number of substitutions per site. * Lactate dehydrogenase, ** Malate/Lactate dehydrogenase, *** Malate dehydrogenase of *Archaeoglobus fulgidus* was characterized as dimeric enzyme [16,24].
